# Biochar Is Not Durable for Remediation of Heavy Metal-Contaminated Soils Affected by Acid-Mine Drainage

**DOI:** 10.3390/toxics10080462

**Published:** 2022-08-09

**Authors:** Junhao Qin, Xi Wang, Jidong Ying, Chuxia Lin

**Affiliations:** 1 College of Natural Resources and Environment, South China Agricultural University, Guangzhou 510642, China; 2 Key Laboratory of Agro-Environment in the Tropics, Ministry of Agriculture of China, Guangzhou 510642, China; 3 Centre for Regional and Rural Futures, Faculty of Science, Engineering and Built Environment, Deakin University, Burwood, VIC 3125, Australia

**Keywords:** heavy metal, biochar, phytoavailability, plant uptake, bioaccessibility, contaminated soil

## Abstract

Biochar is a soil conditioner for enhancing plant growth and reducing plants’ uptake of heavy metals. However, the protonation of biochar surfaces in acid soils can weaken the capacity of biochar to reduce the phytoavailability of soil-borne heavy metals over time. The aim of this study was to test this hypothesis by performing a plant-growth experiment with five harvest cycles to examine the durability of rice-straw biochar for the remediation of an acidic-mine-water-contaminated soil. The application of the biochar significantly reduced the phytoavailability of the heavy metals and inhibited the plant uptake of cationic heavy metals but not anionic Cr. The beneficial effects of the biochar were weakened with the increasing number of harvest cycles caused by the gradual protonation of the biochar surfaces, which resulted in the desorption of the adsorbed heavy metals. The weakening capacity of the biochar to reduce the heavy-metal uptake by the vegetable plants was more evident for Cu, Zn, and Pb compared to Ni and Cd. The experimental results generally confirmed the hypothesis. It was also observed that the bioaccessible amount of various metals in the edible portion of the vegetable was also reduced as a result of the biochar application.

## 1. Introduction

The biochar produced from the pyrolysis of organic matter can be used as a soil amendment to promote plant growth and impede plants’ uptake of heavy metals from contaminated soils [1,2,3]. This is particularly beneficial for the production of vegetables, especially leafy vegetables, which tend to easily take up heavy metals from horticultural soils, which are commonly contaminated by heavy metals due to their relatively frequent exposure to various contamination sources [4,5].

Plants can only take up heavy metals dissolved in soil solutions. The dissolution of soil-borne heavy metals is predominantly driven by soil acidification caused by the generation of either inorganic acids, such as sulfuric acid from the oxidation of sulfide minerals [6,7,8], or the organic acids released from plant roots [9,10]. Biochar materials frequently have an alkaline pH, which allows the neutralization of soil acidity. Biochar materials also have a large specific area due to their highly porous nature. The carboxylic, phenolic, hydroxyl, carbonyl, and quinone groups present on biochar surfaces make them capable of adsorbing heavy metals [11].

Increasing research has been undertaken to investigate the effects of biochar materials on the prevention of plants’ uptake of heavy metals from soils and improvements in plant-growth performance in recent years [12]. Khan et al. [3] found that the application of poplar wood and sugarcane bagasse biochars to mine soils reduced the uptake of chromium and lead by lettuce. Sui et al. [13] showed the effects of biochar on the uptake of cadmium and lead by wheat. Nzediegwu et al. [14] demonstrated that the levels of Cd and Zn in potato flesh were significantly reduced by soil amendment with biochar. Biochar application also increased plants’ water-use efficiency and biomass production while reducing Cu concentration in *Brassica juncea* L. [15]. Medyńska-Juraszek [16] suggested that the effects of biochar on the plant uptake of heavy metals take place through interactions in the rhizosphere, where organic acids are released from plant roots. It has been shown that low-molecular-weight organic acids can alter the surface charge of biochar and affect its capacity to adsorb heavy metals [17]. So far, research work focusing on the durability of biochar materials in terms of their capacity to reduce the phytoavailability of soil-borne heavy metals is limited. This represents a knowledge gap that needs to be closed to improve the evaluation of the cost-effectiveness of using biochar to remediate contaminated soils.

The acidic water generated from mining operations involving coal and metal ores that contain sulfide minerals is a common source of heavy metals in aquatic ecosystems and agricultural lands [18,19]. For acidic-mine-water-contaminated soils, the strong acidity that is commonly encountered tends to keep a large amount of the mine-water-derived heavy metals in bioavailable forms, which can cause toxicity to plants and soil microbes, which play a crucial role in nutrient cycling [20,21]. Given its alkaline nature, biochar could be an ideal amendment for the remediation of acidic soils resulting from mine-water contamination. The soil application of biochar at a high rate for agricultural production could be cost-prohibitive [22]. Therefore, it is only practical to add biochar to contaminated soils at an economically viable application rate for the production of leafy vegetables. So far, most of the experiments aiming to examine the effects of biochar on enhancing vegetable-plant growth and reducing the plant uptake of heavy metals have been limited to a single cropping cycle for determining the suitable biochar application rate. However, when the added biochar is exposed to the H^+^ present in acidic soils, the protonation of biochar surfaces can take place [23]. Protonated biochar surfaces tend not to favor the adsorption of cationic heavy metals [24,25]. It is therefore possible that the degree of biochar-surface protonation intensifies over time, resulting in the desorption of the previously adsorbed heavy metals. This could reduce the capacity of the biochar to immobilize soil-borne heavy metals and, thus, make the beneficial effects of biochar unsustainable.

It is hypothesized that biochar is not durable when its application rate is determined based on the plant-growth performance in the first cropping cycle. This study aimed to test this hypothesis by examining the performance of a selected biochar for impeding heavy-metal uptake by a vegetable plant grown in heavily contaminated soils over a period of time covering multiple harvest cycles of the above-ground plant part to evaluate the durability of the biochar for its intended beneficial use.

## 2. Materials and Methods

### 2.1. The Soil Material

A typical mine-water-contaminated soil was used for the greenhouse experiment. The area from which the soil sample was taken experienced land irrigation with river water affected by acid-mine drainage for decades [26,27]. The soil had a pH of 4.30 and contained multiple heavy metals. The concentrations of Cd, Cr, Cu, Ni, Pb, and Zn were 0.49, 58.7, 246, 15.0, 171, and 256 mg/kg, respectively. The soil contained 14.7 g/kg of organic carbon and had an EC value (electrical conductivity) of 0.723 dS/m (Table S1).

### 2.2. The Test-Vegetable-Plant Species

A common vegetable species, *Gynura cusimbua*, was selected as the test plant in the microcosm experiment. Seed germination and seedling development were conducted using the Murashige and Skoog (MS) basal medium prior to transplanting of the seedlings to the growth chambers.

### 2.3. The Biochar Material

The rice-straw-derived biochar used in the growth experiment was purchased from a commercial source. It was synthesized at 600 °C (pyrolysis temperature), according to the manufacturer’s instructions. It had a pH of 9.94 and a specific surface area of 37.9 m^2^/g (determined by a specific-surface-area and porosity analyzer, Gemini VII 2390, Micromeritics, Norcross, GA, USA). Details of physicochemical characteristics of the biochar material are provided in Table S1.

### 2.4. Microcosm Experiment

A 150-day greenhouse experiment was conducted to observe the changes in phytoavailable heavy metals in the soils and plant-tissue-borne heavy metals. Pre-experiment test suggested that an economically viable application rate of 2.5% (biochar/soil) allowed healthy growth of the test-vegetable plants from a pot trial with only one harvest. One control and one treatment were set for the multi-harvest experiment. For the treatment, 62.5 g of biochar were thoroughly incorporated into 2.5 kg of the contaminated soil. The soil without added biochar served as the control. The respective soil was placed into a plastic pot (top diameter: 27 cm; height: 20 cm). Prior to the plant-growth experiment, the soils were incubated for 15 days. Twelve plant seedlings were then transplanted to each pot. Field capacity was maintained for the soil-moisture content during the entire period of the experiment.

Five harvest cycles (30 days per cycle) were performed during the experiment. At each of the 5 harvest times, one representative plant (aerial portion plus root portion) was removed from each pot. For the remaining plants in each pot, only the aerial portion (edible part) was harvested. The plants were then allowed to re-grow for the next 30 days prior to subsequent harvest. In each pot, chemical fertilizer was added to the soil with an application rate of 4.5 g/pot at the beginning of the experiment and following the first, second, third, and fourth harvest events. 

In the laboratory, the plant samples collected at each harvest time were washed with tap water, followed by deionized water. The whole plant was then separated into aerial portion and root portion. The plant portions were oven-dried to constant weight [23]. Plant powders were prepared using a pestle and mortar. For determination of bioaccesible heavy metals, the edible vegetable-plant portion was cut into small pieces and mixed thoroughly. The plant mixture was then ground with a pestle and mortar before being split into two equal parts and frozen prior to analysis.

### 2.5. Analytical Methods

Titratable acidity of the soils was measured by titrating an aliquot of 1:5 (soil:1 M KCl) extract using 0.01 M standardized NaOH solution. Water-extractable heavy metals and NH_4_Cl-extractable heavy metals were determined from 1:5 (soil: water) extracts and 1:5 (soil: 1 M NH_4_Cl) extracts, respectively. The concentrations of various heavy metals were determined by inductively coupled plasma mass spectrometry (ICP-MS, Agilent 7700). The soil-borne heavy metals were also determined by ICP-MS after digesting 0.15 g of the soil with a mixed solution of HNO_3_, HF, and H_2_O_2_ in a microwave digester [23].

Heavy metals in the edible plant tissues were determined by ICP-MS after digestion of the oven-dried plant powder (0.10 g) with a mixed solution of HNO_3_ and H_2_O_2_ in a microwave digester [23]. To determine the bioaccessible heavy metals in the vegetable edible portion, a modified unified BARGE method was adopted [28]. 

### 2.6. Quality Assurance and Quality Control

Certified reference materials were used for determination of various heavy metals in the soil samples (GBW07407) and the plant-tissue samples (GBW(E)100349), respectively. The recovery rates for Cr, Ni, Cu, Zn, Cd, and Pb were 86.5%, 90.6%, 105.2%, 95.7%, 89.9%, and 104.8%, respectively. Triplicated experiment was performed in this study. Repeatability analysis shows that the RSD (relative standard deviation) was 12.2%, 9.90%, 8.63%, 6.38%, 3.12%, and 9.32% for the total Cr, Ni, Cu, Zn, Cd, and Pb, respectively, in the plant tissue, 8.83%, 10.5%, 5.86%, 5.57%, 7.72%, and 8.85%, respectively, for the gastric (G) phase, and 7.82%, 6.51%, 7.69%, 5.76%, 6.26%, and 8.48%, respectively, for the gastrointestinal (GI) phase.

### 2.7. Statistical Analysis Method

The experimental data were analyzed using IBM SPSS^®^ Statistics 22.0 software. Analysis of normal distribution and equal variance was performed to test whether each parameter in either the control or the treatment depended on each harvest cycle, and whether each parameter in the same harvest cycle depended on the control and the treatment. If the equal variances were assumed, one-way analysis of variance (ANOVA) with Duncan’s multiple range test was used to compare the means of each parameter for the 5 harvest cycles, while significant differences between the control and the treatment for each parameter were determined by an independent sample t-test at 0.05 level or 0.01 level.

### 2.8. Assessment Criteria

Bioaccumulation factor (BAF) was used to evaluate the degree of heavy-metal uptake by plant root:

BAF = HMroot/HMsoil(1)

The translocation of a heavy metal from root portion to aerial portion of the plant was evaluated by translocation factor (TF):

TF = HMaerial/HMroot(2)

where HMsoil, HMroot, and HMaerial denote the total heavy-metal concentrations in the soil, root, and aerial portion, respectively.

The bioaccessible concentration of a heavy metal in the edible vegetable portion and the bioaccessibility of that heavy metal were calculated as follows:

Bioaccessible heavy metal (mg/kg) = (Con × Vol)/Wt(3)

Bioaccessibility (%) = bioaccessible HM/total HMplant × 100(4)

where Con and Vol are the concentration (µg/mL) of a heavy metal in the simulated gastric (G) or gastrointestinal (GI) solutions and the volume (mL) of the simulated G or GI solutions, respectively. Wt (g) denotes the fresh biomass of the vegetable sample used to determine the bioassessible heavy metals (Bioaccessible HM). Total HMplant is the total heavy metal in the edible vegetable portion. Bioaccessible HM and total HMplant were all expressed on a fresh-weight basis.

## 3. Results

### 3.1. Effects of Biochar on Soil Acidity and Phytoavailable Heavy Metals

**Figure 1:**
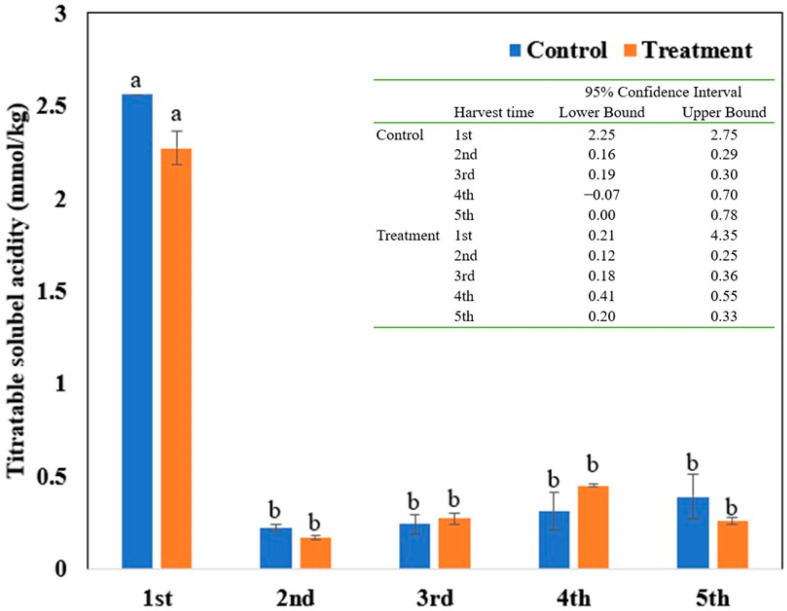
Temporal variation in soil titratable acidity in the control and the treatment. The data are presented as mean ± standard error (n = 3) with different letters above the bars for either the control or the treatment indicating significant differences at *p* < 0.05. The 95% confidence intervals for different harvest cycles in the control and the treatment are also provided.

At the time of the first harvest, the titratable acidity in the soil amended with biochar (1.10 ± 0.08 mmol/kg) was significantly lower compared to the control (2.50 ± 0.00 mmol/kg). For the unamended soil, the titratable acidity tended to decrease from the first harvest time to the second harvest time and then to remain at a level below 1 mmol/kg for the later harvest times. For the amended soil, no significant difference in titratable acidity was observed among the different harvest times, except for the second harvest time, when the titratable acidity was significantly lower relative to the other harvest times (Figure 1).

No significant difference in the water-extractable Cr was observed between the unamend soil (control) and the amended soil, except at the first and third harvest times, when the amended soil had a higher water-extractable Cr concentration relative to the control soil. The NH_4_Cl-extratable Cr showed no significant differences between the control soil and the amended soil either, except at the fourth harvest time, when the concentration of the NH_4_Cl-extratable Cr was higher in the amended soil compared to the control soil (Table 1).

Mixed results were found for the water-extractable Ni, with the first and fourth harvest times showing a higher (significantly at *p* < 0.05) Ni concentration in the amended soil than in the unamended control soil, the second and third harvest times showing lower (significantly at *p* < 0.05) Ni concentrations in the biochar-treated soil compared to the unamended control soil, and the fifth harvest time showing no significant difference (*p* > 0.05) in Ni concentration between the unamended soil and the amended soil. For the NH_4_Cl-extratable Ni, there was a significantly (*p* < 0.05) lower concentration in the biochar-treated soil than in the unamended soil at all of the five harvest times (Table 1).

For the water-extractable Cu, it was clear that the concentration in the amended soil was lower relative to the control soil for the first three harvest cycles, and the opposite was observed for the last two harvest cycles. For the NH_4_Cl-extratable Cu, the concentration in the amended soil was lower compared to the unamended control soil for the first two harvest cycles, and the opposite was observed for the last two harvest cycles, with no significant difference in the NH_4_Cl-extratable Cu observed between the unamended control soil and the amended soil (Table 1).

Significant differences in the water-extractable Zn were observed between the unamended control soil and the biochar-treated soil, with the first, fourth, and fifth harvest times featuring higher concentrations in the amended soil relative to the unamended control soil, while the opposite was observed for the second and third harvest cycles. There was no clear variation trend in the water-extractable Zn from the first to the fifth harvest time for both the control soil and the biochar-amended soil. For the NH_4_Cl-extratable Zn, the concentration in the biochar-treated soil was always higher relative to the unamend control soil, and always at significant levels, except at the fifth harvest time (Table 1).

No significant difference in the water-extractable Cd was observed, except at the fifth harvest time, when the water-extractable Cd in the amended soil was higher compared to the unamended soil. There was no clear temporal variation in the water-extractable Cd for either the unamended soil or the amended soil. For the NH_4_Cl-extratable Cd, the concentration in the amended soil was significantly lower compared to the unamended control soil for all the harvest cycles. While there was no clear variation trend in the NH_4_Cl-extratable Cd from the first to the fifth harvest time for the unamended soil, the NH_4_Cl-extratable Cd tended to decrease with increasing numbers of harvest cycles for the biochar-treated soil (Table 1).

The water-extractable Pb in the biochar-treated soil was significantly lower compared to the control soil at the first three harvest times, but the opposite was observed for the last harvest times. There was no clear temporal variation trend in the water-extractable Pb for the unamended control soil and the biochar-treated soil. For the NH_4_Cl-extratable Pb, only the first harvest cycle showed a lower Pb concentration in the biochar-treated soil compared to the unamended control soil. For the other harvest cycles, either the opposite was the case, or no significant difference was observed in the NH_4_Cl-extratable Pb between the unamended control soil and the biochar-treated soil (Table 1).

### 3.2. Plant-Tissue-Borne Heavy Metals at Different Harvest Times

#### 3.2.1. Chromium

There was no significant difference in the root-borne Cr between the vegetable plant grown in the unamend soil (VP-unamended soil) and the vegetable plant grown in the amended soil (VP-amended soil), except at the third harvest time, when the root-borne Cr in the VP-amended soil was significantly lower relative to the VP-unamended soil. However, for the aerial portion, the concentration of the plant-tissue-borne Cr in the VP-amended soil was significantly lower compared to the VP-unamended soil, except at the third harvest time, when the opposite was observed (Table 2).

#### 3.2.2. Nickel

The root-borne Ni in was significantly lower in the VP-amended soil than in the VP-unamended soil at any of the five harvest times. Furthermore, the root-borne Ni tended to decrease as the number of the harvest cycle increased for both the VP-unamended soil and the VP-amended soil. For the above-ground portion, mixed results were observed: the VP-unamended soil and the VP-amended soil showed no significant difference in root-borne Ni for the first and third harvest cycles; the root-borne Ni in the VP-amended soil was significantly lower relative to the VP-unamended soil for the second harvest cycle; and at the last two harvest times, the root-borne Ni in the VP-amended soil was significantly higher relative to the VP-unamended soil (Table 2).

#### 3.2.3. Copper

The root-borne Cu in the VP-amended soil was lower (significantly at *p* < 0.05) relative to the VP-unamended soil for the first to third harvest cycles, while the VP-unamended soil and the VP-amended soil showed no significant differences in the root-borne Cu for the last two harvest cycles. A trend was observed in which the root-borne Cu increased as the number of harvest cycles increased in the VP-amended soil, while there was no clear temporal variation in the root-borne Cu in the VP-unamended soil. For the above-ground portion, the Cu in the VP-amended soil was higher (significantly at *p* < 0.05) relative to the VP-unamended soil for the first and second harvest cycles, but the control and the treatment showed no significant difference in Cu for the last three harvest cycles (Table 2).

#### 3.2.4. Zinc

The root-borne Zn in the VP-amended soil was lower (significantly at *p* < 0.05) compared to the VP-unamended soil for the first three harvest cycles, but the VP-unamended soil and the VP-amended soil exhibited no significant (*p* > 0.05) differences in the root-borne Zn between for the last two harvest cycles. For the above-ground portion, the Zn in the VP-amended soil was lower (significantly at *p* < 0.05) compared to the VP-unamended soil for the first two harvest cycles, but the VP-unamended soil and the VP-amended soil showed no significant differences in Zn for the last three harvest cycles (Table 2).

#### 3.2.5. Cadmium

The VP-unamended soil and the VP-amended soil showed no significant differences in the root-borne Cd, except in the third harvest cycle, when the root-borne Cd in the VP-amended soil was significantly lower compared to the VP-unamended soil. The concentrations of the root-borne Cd tended to be higher in the earlier harvest cycles than in the later harvest cycles for both the VP-unamended and the VP-amended soils. For the above-ground portion, there was no significant difference in Cd between the VP-unamended soil and the VP-amended soil, except for the fourth harvest cycle, which showed higher plant tissue Cd in the VP-amended soil than in the VP-unamended soil. There was no clear temporal variation in the plant-tissue Cd for either the VP-unamended or the VP-unamended soils (Table 2).

#### 3.2.6. Lead

The root-borne Pb in the VP-amended soil was lower (significantly at *p* < 0.05) compared to the VP-unamended soil for the first three harvest cycles, but the VP-unamended and VP-amended soils showed no significant differences in the root-borne Pb for the later harvest cycles. The root-borne Pb tended to increase as the number of harvest cycle increased for the VP-amended soil, while no clear trend was observed for the VP-unamended soil. For the above-ground portion, similar trends to the below-ground portion were observed; the Pb in the VP-amended soil was also significantly lower relative to the VP-unamended soil for the first three harvest cycles, but the VP-unamended soil and the VP-amended soil showed no significant differences in root-borne Pb for the later harvest cycles; the root-borne Pb tended to increase as the number of harvest cycles increased for the VP-amended soil, while no clear trend was established for the VP-unamended soil (Table 2).

### 3.3. Bioaccessible Heavy Metals in the Edible Vegetable Portion

The bioaccessible amounts of various heavy metals in the edible vegetable portion was lower in the VP-amended soil compared to the VP-unamended soil for the gastric phase and gastrointestinal phase (*p* < 0.05), although it was not statistically significant for the Cd at a few of the harvest times (Table 3). The concentrations of bioaccessible heavy metals varied with the harvest time but showed no clear increasing or decreasing trend with increasing numbers of harvest cycles.

## 4. Discussion

The addition of alkaline biochar to the acidic soil caused a decrease in the titratable acidity at the first harvest time (Figure 1). This was accompanied by a reduction in the water-extractable Cu and Pb (Table 1), reflecting the immobilization of the soluble Cu and Pb, possibly through hydrolysis or adsorption by the biochar [29,30,31,32]. For example, hydrolysis resulted in the transformation of the soluble Cu^2+^ into Cu(OH)^+^ and, eventually, precipitation as insoluble Cu(OH)_2_:

Cu^2+^ + OH^−^ → Cu(OH)^+^ (5)

Cu(OH)^+^ → Cu(OH)_2_ (6)

Following the addition of the biochar to the soil, the negatively charged surfaces allowed the adsorption of cationic heavy metals to the biochar.

[Biochar]^2^^−^ + 2Cu(OH)^+^ → [Biochar]^2^^−^ − 2Cu(OH)^+^ (7)

[Biochar]^2^^−^ + Pb^2+^ → [Biochar]^2^^−^ − Pb^2+^ (8)

The application of the biochar also resulted in a decrease in the NH_4_Cl-extractable heavy metals, except for the Cr. The NH_4_Cl-extractable fraction of a heavy metal includes the exchangeable form of that heavy metal in addition to its soluble form. Therefore, it represents the major phytoavailable pool of heavy metals in soils [33,34]. Under the predominantly oxidizing conditions encountered in the soils in the growth experiment, Cr was likely to be present in oxyanionic forms (CrO_4_^2−^ or Cr_2_O_7_^2−^) [35,36]. Unlike cationic heavy metals, anionic Cr was not immobilized via hydrolysis or adsorption to negatively charged biochar surfaces. This explains why no reduction was observed in the soluble and exchangeable Cr from the reduced soil acidity after the biochar application. The general tendency of the concentrations of the cationic heavy metals in the amended soil, particularly Cu, Zn, and Pb, was lower compared to the unamended soil at the earlier harvest times, which suggests that the addition of the biochar had the effect of immobilizing these heavy metals. However, the protonation of the biochar surfaces took place over time, since the soil had a pH of around 4. This might have led to the replacement of the adsorbed heavy metals by H ^+^, as shown by the following example:

[Biochar]^2^^−^ − Cu^2+^ + 2H^+^ → [Biochar]^2^^−^ − 2H^+^ + Cu^2+^ (9)

This process could also have been enhanced by the generation of organic acid in the rhizosphere from plant-root exudation [23,37]. 

**Figure 2:**
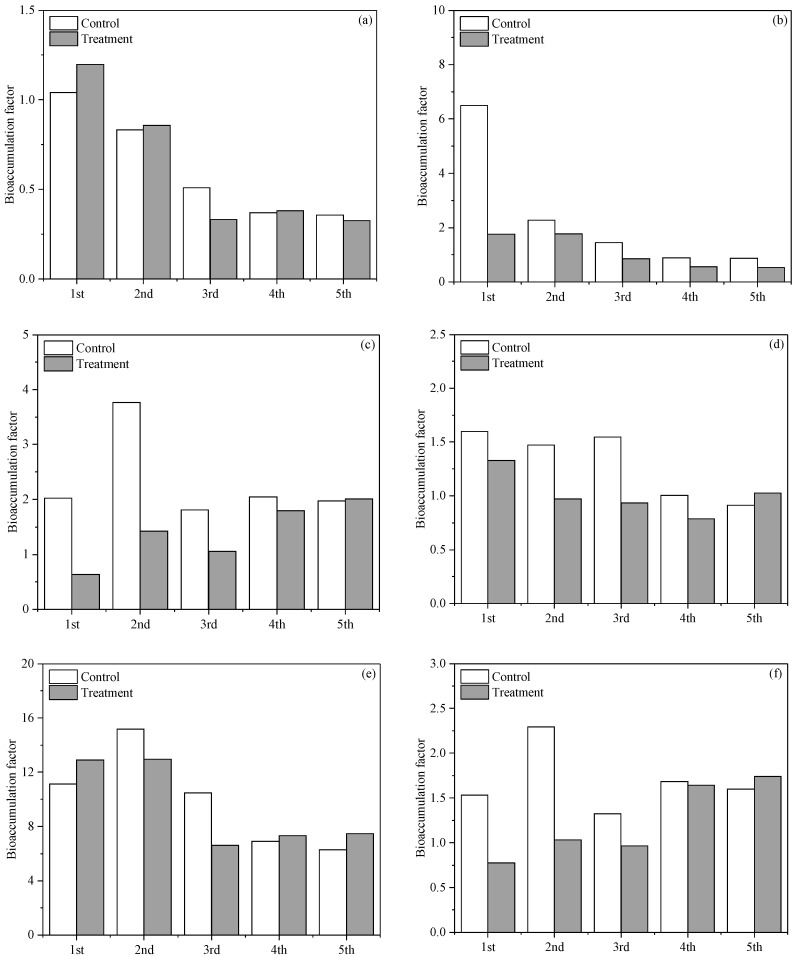
Bioaccumulation factor (BAF) of Cr (**a**), Ni (**b**), Cu (**c**), Zn (**d**), Cd (**e**), and Pb (**f**) for the *Gynura cusimbua* at different harvest times.

The relatively highly soluble Cu and Pb in the soil at the last two harvest times also resulted in higher concentrations of the root-borne Cu and Pb at these two harvest times, reflecting the enhanced uptake of these two heavy metals by the roots, which might have affect the growth of the plants [38,39,40]. From Figure 2, it can be seen that the BAF of the Cu, Zn, and Pb showed a similar pattern, in which the BAF in the unamended soil tended to be higher relative to the amended soil at the earlier harvest times, while the BAF values in the control and the biochar treatment were very close to each other at the later harvest times. This suggests that the effect of the added biochar on the prevention of the uptake of these three heavy metals was reduced over time. Unlike the three aforementioned heavy metals, the BAF of the Cr showed no clear temporal variation, and in most of the harvest times, the BAF values of the control and the biochar treatment were very close, suggesting that the added biochar had no marked effects on the uptake of the soil-borne Cr by the plant roots. This is attributable to the weak effect of the added biochar on the Cr’s phytoavaibility. The BFA of the Ni in the control was higher relative to the biochar treatment for all five harvest cycles, indicating that the reduction in the plant uptake of Ni by the biochar lasted for the entire duration of the experiment. Mixed results were obtained for the BAF of the Cd, although it seems that the capacity of the added biochar to inhibit the uptake of Cd by the roots was weakened at the last two harvest times.

**Figure 3:**
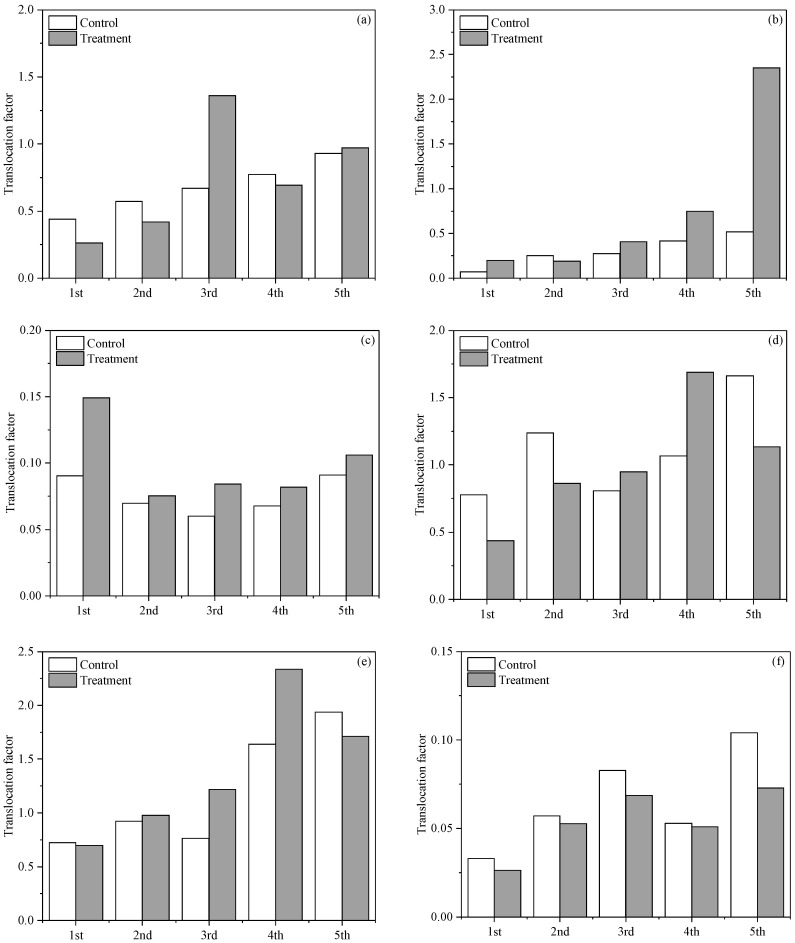
Translocation factor (TF) of Cr (**a**), Ni (**b**), Cu (**c**), Zn (**d**), Cd (**e**), and Pb (**f**) for the *Gynura cusimbua* at different harvest times.

With regard to the root–aerial-portion translocation of the heavy metals, different distribution patterns of TF were observed for the different heavy metals across the five harvest times. The TF for a heavy metal could be higher or lower in the control than in the biochar treatment, depending on the harvest time, on a random basis (Figure 3). This suggests that the biochar application had insignificant effects on the root–shoot translocation of the heavy metals. It is interesting to note that the Cu and Pb showed much lower TF compared to the other heavy metals. This is in agreement with the findings by Mirecki et al. [41]. By contrast, the TF in the Cd tended to be greater than in the other heavy metals. The high root–shoot translocation rate is widely recognized [42]. The variation in TF values among the different heavy metals may be attributed to the differential solubilities and tendencies toward compartmentalization of individual heavy metals in the plant vascular system [43].

While, on most occasions, the heavy-metal bioaccessibility in gastric phase was lower in the added-biochar treatment compared to the control, the opposite was observed for some occasions, which were not necessarily during the last harvest time (Figure S1). Therefore, biochar application might or might not have effects on reductions in the bioaccessibility of the edible portion of the vegetable.

There was a marked difference in bioaccessibility in the G phase among the different heavy metals, and at different times of harvest, indicating the complexity of the factors that affect the bioaccessbility of heavy metals contained in the edible vegetable portion. In general, the bioaccessibility of the plant-borne Cr and Pb was lower than that of the heavy metals, some of which displayed bioaccessibility greater than 90%.

In general, there was a high level of similarity between the G-phase bioaccessibity and the GI-phase bioaccessibility, showing that the plant-borne Cr and Pb had lower bioaccessibility than the other heavy metals (Figure S2). This appears to suggest that the intake of the heavy metals associated with consumption of the vegetable was more likely to occur during the stage of gastric digestion.

## 5. Conclusions

The application of biochar to the acidic-mine soil resulted in a reduction in the phytoavailability of the heavy metals, which impeded the plant uptake of cationic heavy metals, but not of the anionic Cr. However, these beneficial effects of biochar were weakened as the number of harvest cycles increased after the negatively charged surfaces of biochar were gradually protonated under acidic soil conditions. This led to the desorption of the previously adsorbed heavy metals. The weakening capacity of the biochar to impede the uptake of heavy metals by the vegetable plant was more evident for the Cu, Zn, and Pb than for the Ni and Cd. The bioaccessible amounts of heavy metals in the edible vegetable portion were also reduced due to the biochar application.

## Figures and Tables

**Table 1:** Water-extractable and NH_4_Cl-extratable heavy metals in the soil at different harvest times during the period of the greenhouse experiment.

		Water-Extractable		NH_4_Cl-Extratable	
Element	Harvest Time	Control	Treatment	Control	Treatment
Cr	1st	0.004 ± 0.001a	0.015 ± 0.002a **	0.009 ± 0.000b	0.013 ± 0.002b
	2nd	0.002 ± 0.000bc	0.003 ± 0.000b	0.014 ± 0.002a	0.011 ± 0.001b
	3rd	0.001 ± 0.000c	0.004 ± 0.000b **	0.015 ± 0.001a	0.021 ± 0.004a
	4th	0.003 ± 0.000ab	0.003 ± 0.000b	0.005 ± 0.000c	0.013 ± 0.002b *
	5th	0.002 ± 0.000c	0.002 ± 0.000b	0.012 ± 0.001ab	0.010 ± 0.000b
Ni	1st	0.073 ± 0.001c	0.118 ± 0.001ab **	0.585 ± 0.012d	0.533 ± 0.008b *
	2nd	0.092 ± 0.006b	0.074 ± 0.003c *	0.648 ± 0.011c	0.602 ± 0.015a *
	3rd	0.127 ± 0.007a	0.099 ± 0.003b *	0.708 ± 0.008b	0.621 ± 0.007a **
	4th	0.086 ± 0.007bc	0.133 ± 0.011a *	0.829 ± 0.005a	0.538 ± 0.009 **
	5th	0.074 ± 0.001c	0.073 ± 0.008c	0.614 ± 0.015cd	0.530 ± 0.009b **
Cu	1st	0.443 ± 0.018b	0.257 ± 0.020bc **	7.201 ± 0.069a	5.423 ± 0.268cd **
	2nd	0.193 ± 0.083c	0.167 ± 0.016c	6.187 ± 0.312b	4.931 ± 0.110d *
	3rd	0.632 ± 0.045a	0.299 ± 0.005bc **	6.147 ± 0.263b	6.877 ± 0.228b
	4th	0.292 ± 0.004c	1.553 ± 0.097a **	4.334 ± 0.093c	8.149 ± 0.019a **
	5th	0.262 ± 0.009c	0.378 ± 0.009b **	4.022 ± 0.229c	5.597 ± 0.068c *
Zn	1st	4.191 ± 0.119b	5.613 ± 0.405b *	14.25 ± 0.230b	12.57 ± 0.372a *
	2nd	4.371 ± 0.100b	3.367 ± 0.155c **	16.66 ± 0.710b	12.61 ± 0.343a **
	3rd	6.099 ± 0.131a	5.253 ± 0.078b **	16.35 ± 1.023b	13.12 ± 0.605a *
	4th	4.039 ± 0.185b	7.629 ± 0.145a **	23.50 ± 0.808a	13.93 ± 0.069a **
	5th	3.313 ± 0.022c	4.008 ± 0.128c **	15.66 ± 1.321b	13.45 ± 0.754a
Cd	1st	0.024 ± 0.002ab	0.020 ± 0.001d	0.135 ± 0.002b	0.111 ± 0.004a **
	2nd	0.021 ± 0.001b	0.018 ± 0.001d	0.134 ± 0.009b	0.104 ± 0.006a *
	3rd	0.039 ± 0.001a	0.033 ± 0.001b	0.157 ± 0.016b	0.100 ± 0.003ab*
	4th	0.033 ± 0.011ab	0.045 ± 0.002a	0.223 ± 0.006a	0.103 ± 0.002ab **
	5th	0.020 ± 0.001b	0.027 ± 0.001c **	0.143 ± 0.006b	0.091 ± 0.003b **
Pb	1st	0.075 ± 0.006a	0.036 ± 0.001b **	22.436 ± 0.346a	18.044 ± 0.669c **
	2nd	0.052 ± 0.002bc	0.039 ± 0.002b *	19.020 ± 0.553b	18.190 ± 0.525bc
	3rd	0.077 ± 0.001a	0.050 ± 0.004b **	19.287 ± 0.151b	20.252 ± 0.642a
	4th	0.061 ± 0.004b	0.282 ± 0.021a **	11.782 ± 0.508d	19.704 ± 0.090ab **
	5th	0.047 ± 0.001c	0.060 ± 0.002b **	13.729 ± 0.158c	12.524 ± 0.332d

All values are presented as mean ± standard error (n = 3) and means with different letters in the same column for each heavy metal are significantly different (*p* < 0.05). Independent-sample t-test was used to determine whether the two mean values obtained for the control and the treatment differed significantly. “*” indicates significant difference at *p* < 0.05 between the control and the treatment for each harvest time. “**”indicates significant difference *p* < 0.01 between the control and the treatment for each harvest time.

**Table 2:** Concentrations (mg/kg) of various heavy metals in the above-ground and below-ground parts of Gynura cusimbua in the growth experiment.

		Above-Ground Portion		Below-Ground Portion	
Element	Harvest Time	Control	Treatment	Control	Treatment
Cr	1st	26.88 ± 1.60a	18.47 ± 0.89bc *	61.13 ± 5.71a	70.37 ± 5.95a
	2nd	28.09 ± 3.28a	21.02 ± 0.99b *	48.93 ± 7.03a	50.24 ± 17.81a
	3rd	20.06 ± 1.48b	26.60 ± 2.47a	29.94 ± 1.53b	19.55 ± 1.91b *
	4th	16.82 ± 1.88b	15.58 ± 0.78c **	21.75 ± 2.13b	22.41 ± 0.94b
	5th	19.47 ± 0.24b	18.58 ± 0.05bc *	20.95 ± 2.64b	19.12 ± 2.45b
Ni	1st	6.80 ± 0.59b	5.25 ± 1.33b	97.50 ± 2.40a	26.25 ± 3.52a **
	2nd	8.58 ± 0.31a	5.00 ± 0.32b **	34.02 ± 1.91b	26.52 ± 0.86a *
	3rd	5.96 ± 0.52b	5.26 ± 0.94b	21.77 ± 2.74c	12.95 ± 1.73b
	4th	5.53 ± 1.78b	6.32 ± 0.10b *	13.36 ± 2.47d	8.45 ± 2.22b
	5th	6.73 ± 0.75b	18.50 ± 1.66a **	13.01 ± 1.36d	7.86 ± 0.36b *
Cu	1st	44.89 ± 4.17b	23.43 ± 2.41bc *	497.58 ± 28.61b	157.00 ± 34.61c **
	2nd	64.55 ± 3.71a	26.41 ± 0.75bc **	926.40 ± 24.22a	350.42 ± 35.91ab **
	3rd	26.60 ± 1.18c	21.78 ± 3.12c	444.27 ± 61.64b	258.80 ± 85.40bc
	4th	34.00 ± 1.85c	36.04 ± 8.33b	503.31 ± 98.12b	440.55 ± 23.05ab
	5th	44.11 ± 3.51b	52.27 ± 0.75a	484.79 ± 39.48b	493.62 ± 70.50a
Zn	1st	316.83 ± 30.43b	148.39 ± 11.63b **	408.69 ± 13.87a	340.32 ± 18.31a *
	2nd	466.71 ± 25.28a	214.41 ± 22.30ab **	377.43 ± 39.65a	248.36 ± 18.30b *
	3rd	318.68 ± 71.63b	226.72 ± 30.23ab	395.56 ± 29.92a	239.06 ± 18.06b *
	4th	273.78 ± 20.13b	339.31 ± 75.92a	257.16 ± 21.85b	200.84 ± 10.25b
	5th	387.65 ± 19.05ab	297.80 ± 58.23ab *	233.04 ± 8.81b	262.70 ± 42.50b
Cd	1st	3.94 ± 0.54b	4.41 ± 0.20bc	5.46 ± 1.08ab	6.32 ± 0.13a
	2nd	6.87 ± 0.36a	6.21 ± 0.36b	7.44 ± 2.14a	6.35 ± 0.65a
	3rd	3.91 ± 0.06b	3.95 ± 0.37c	5.13 ± 0.37ab	3.24 ± 0.29b *
	4th	5.55 ± 0.35a	8.36 ± 0.25a **	3.39 ± 0.27b	3.58 ± 0.45b
	5th	5.97 ± 0.79a	6.27 ± 1.31b	3.08 ± 0.09b	3.66 ± 0.62b
Pb	1st	8.65 ± 0.36d	3.49 ± 0.25d **	262.32 ± 28.48b	132.11 ± 25.02b *
	2nd	22.45 ± 1.21b	9.31 ± 0.05c **	392.43 ± 28.48a	176.44 ± 15.13b **
	3rd	18.70 ± 1.10c	11.34 ± 0.90bc **	226.12 ± 34.38b	165.40 ± 29.20b
	4th	15.25 ± 0.65c	14.27 ± 0.58b	288.00 ± 58.29ab	280.80 ± 16.72a
	5th	28.43 ± 1.77a	21.69 ± 2.23a	273.44 ± 24.61ab	298.00 ± 24.05a

All values are presented as mean ± standard error (n = 3) and means with different letters in the same column for each heavy metal are significantly different (*p* < 0.05). Independent-sample t-test was used to determine whether the two mean values obtained for the control and the treatment differed significantly. “*” indicates significant difference at *p* < 0.05 between the control and the treatment for each harvest time. “**” indicates significant difference *p* < 0.01 between the control and the treatment for each harvest time.

**Table 3:** Concentration (mg/kg on a fresh weight basis) of bioaccessible heavy metals in the edible portion of *Gynura cusimbua*.

		Gastric Phase		Gastrointestinal Phase	
Element	Harvest Time	Control	Treatment	Control	Treatment
Cr	1st	0.14 ± 0.00b	0.03 ± 0.00c **	0.12 ± 0.01b	0.05 ± 0.00b *
	2nd	0.39 ± 0.02a	0.09 ± 0.00a **	0.72 ± 0.05a	0.05 ± 0.00b **
	3rd	0.15 ± 0.00b	0.08 ± 0.01a **	0.14 ± 0.00b	0.09 ± 0.01a **
	4th	0.14 ± 0.00b	0.06 ± 0.00b **	0.19 ± 0.01b	0.08 ± 0.00a **
	5th	0.16 ± 0.00b	0.08 ± 0.00ab **	0.11 ± 0.00b	0.09 ± 0.00a **
Ni	1st	0.30 ± 0.02b	0.09 ± 0.01b **	0.35 ± 0.02bc	0.11 ± 0.00d **
	2nd	0.39 ± 0.02a	0.24 ± 0.03a *	0.38 ± 0.00b	0.17 ± 0.01c **
	3rd	0.34 ± 0.02ab	0.20 ± 0.02a **	0.32 ± 0.01c	0.22 ± 0.01b **
	4th	0.33 ± 0.02ab	0.20 ± 0.00a **	0.35 ± 0.03bc	0.26 ± 0.01a *
	5th	0.37 ± 0.01a	0.23 ± 0.00a **	0.46 ± 0.02a	0.23 ± 0.01b **
Cu	1st	2.61 ± 0.03ab	0.54 ± 0.01d **	2.44 ± 0.03ab	0.74 ± 0.07d **
	2nd	2.55 ± 0.05ab	1.21 ± 0.06b **	2.68 ± 0.05a	0.99 ± 0.02c **
	3rd	1.26 ± 0.07c	1.01 ± 0.04c **	1.61 ± 0.05c	1.14 ± 0.09c **
	4th	1.43 ± 0.05c	1.07 ± 0.05c **	2.12 ± 0.15bc	1.45 ± 0.02b *
	5th	2.71 ± 0.22a	1.82 ± 0.02a *	2.67 ± 0.22a	1.75 ± 0.05a *
Zn	1st	14.89 ± 0.82d	4.57 ± 0.38d **	11.55 ± 0.26c	4.07 ± 0.21d **
	2nd	17.26 ± 0.53c	10.06 ± 0.12c **	21.61 ± 0.77a	8.17 ± 0.26b **
	3rd	19.32 ± 0.14b	12.52 ± 0.21bc **	11.24 ± 0.29c	9.95 ± 0.10a *
	4th	17.04 ± 0.82c	13.18 ± 0.91b *	10.12 ± 0.77c	5.65 ± 0.12c **
	5th	21.02 ± 0.12a	14.96 ± 0.35a **	16.44 ± 0.12b	8.42 ± 0.48b **
Cd	1st	0.24 ± 0.00b	0.13 ± 0.01c **	0.12 ± 0.01d	0.05 ± 0.00e **
	2nd	0.31 ± 0.00a	0.28 ± 0.01a	0.22 ± 0.00b	0.10 ± 0.01c **
	3rd	0.25 ± 0.01b	0.24 ± 0.01b	0.21 ± 0.00b	0.09 ± 0.00d **
	4th	0.31 ± 0.01a	0.22 ± 0.04b	0.19 ± 0.01c	0.14 ± 0.00b *
	5th	0.36 ± 0.01a	0.23 ± 0.00b **	0.28 ± 0.01a	0.22 ± 0.00a **
Pb	1st	0.20 ± 0.01c	0.04 ± 0.00d **	0.02 ± 0.00c	0.01 ± 0.00c *
	2nd	0.80 ± 0.00a	0.14 ± 0.00b **	0.54 ± 0.03a	0.03 ± 0.00b **
	3rd	0.19 ± 0.02c	0.11 ± 0.01c *	0.07 ± 0.00b	0.04 ± 0.00a **
	4th	0.28 ± 0.01b	0.10 ± 0.01c **	0.07 ± 0.01b	0.03 ± 0.01b **
	5th	0.81 ± 0.02a	0.46 ± 0.00a **	0.10 ± 0.00b	0.05 ± 0.00a **

All values are presented as mean ± standard error (n = 3) and means with different letters in the same column for each heavy metal are significantly different (*p* < 0.05). Independent-sample t-test was used to determine whether the two mean values obtained for the control and the treatment differed significantly. “*” indicates significant difference at *p* < 0.05 between the control and the treatment for each harvest time. “**” indicates significant difference *p* < 0.01 between the control and the treatment for each harvest time.

## Data Availability

Not applicable.

## Supplementary Materials

The following supporting information can be downloaded at: https://www.mdpi.com/article/10.3390/toxics10080462/s1. Figure S1: Bioaccessibility (%) of Cr (a), Ni (b), Cu (c), Zn (d), Cd (e), and Pb (f) in the edible portion of the vegetable under simulated gastric conditions at different harvest times. Figure S2: Bioaccessibility (%) of Cr (a), Ni (b), Cu (c), Zn (d), Cd (e), and Pb (f) in the edible portion of the vegetable under simulated gastrointestinal conditions at different harvest times. Table S1: Some major physicochemical characteristics of the biochar and soil used in the greenhouse experiment.
