# Supplementary material for: Biochar Is Not Durable for Remediation of Heavy Metal-Contaminated Soils Affected by Acid-Mine Drainage
Source: Toxics. 2022 Aug 9;10(8):462. doi: 10.3390/toxics10080462 (PMC9416525; doi:10.3390/toxics10080462)
Supplement: Supplementary file 1 [file toxics-10-00462-s001.zip › toxics-1823308-supplementary.pdf]

Supplementary Information

1 Supplementary Table

Table S1. Some major physicochemical characteristics of the biochar and soil used in the greenhouse experiment.

Parameter	Biochar	Soil
**pH**	9.94	4.30
**EC (dS/m)**	8.050	0.723
**BET surface area (m^2/g)**	37.85	
**Zeta potential (mv)**	-60.9	
**Total organic C (g/kg)**	420	14.7
**Total N (g/kg)**	7.9	1.72
**Total P (mg/kg)**	2.35	
**Available P (mg/kg)**	59.23	0.018
**Available K (g/kg)**	9.65	0.023
**Total Cr (mg/kg)**		58.7
**Total Ni (mg/kg)**		15
**Total Cu (mg/kg)**		246
**Total Zn (mg/kg)**		256
**Total Cd (mg/kg)**		0.49
**Total Pb (mg/kg)**		171

2 Supplementary Figures

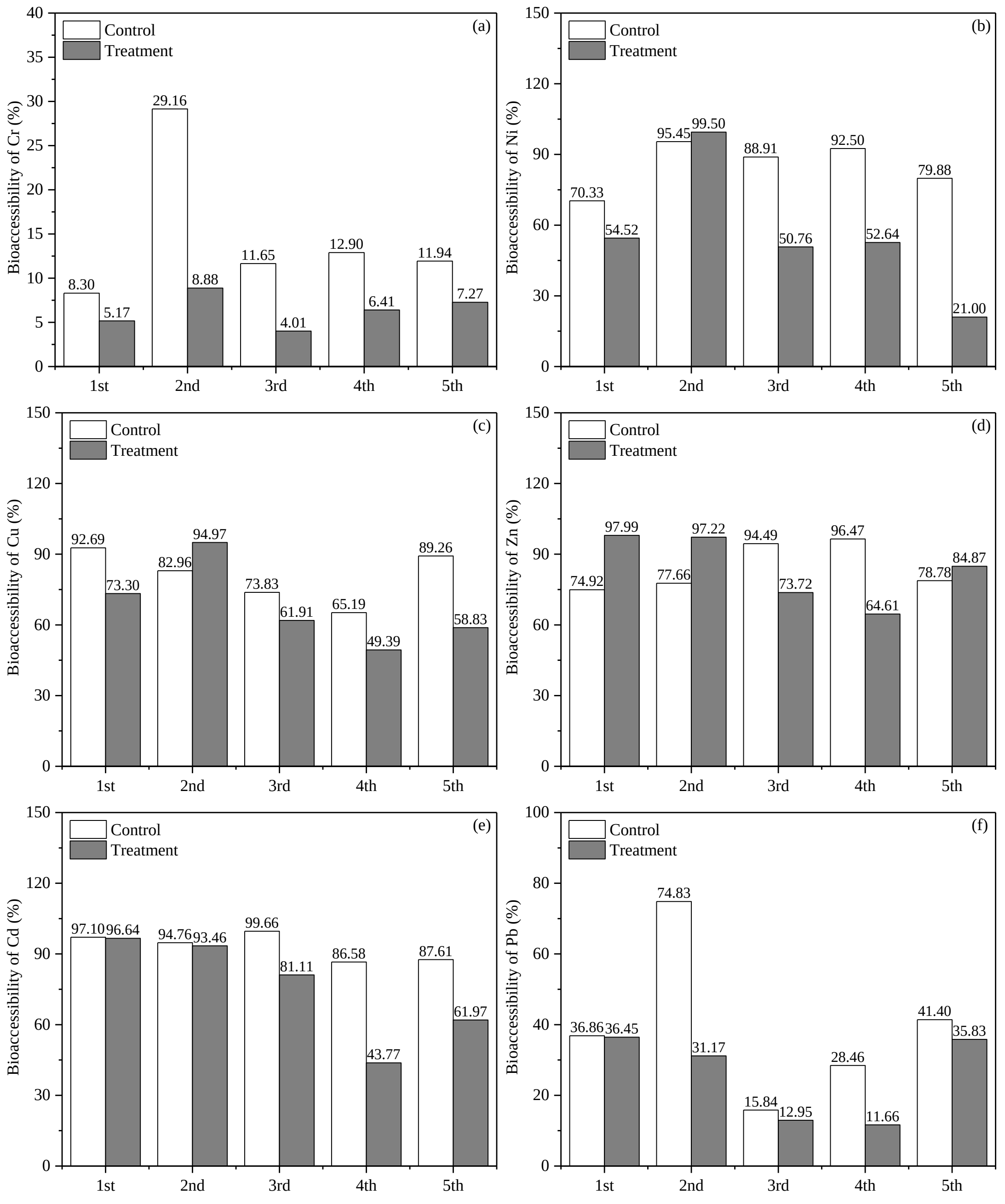

Figure S1. Bioaccessibility (%) of Cr (a), Ni (b), Cu (c), Zn (d), Cd (e) and Pb (f) in the edible portion of the vegetable under simulated gastric conditions at different harvest times.

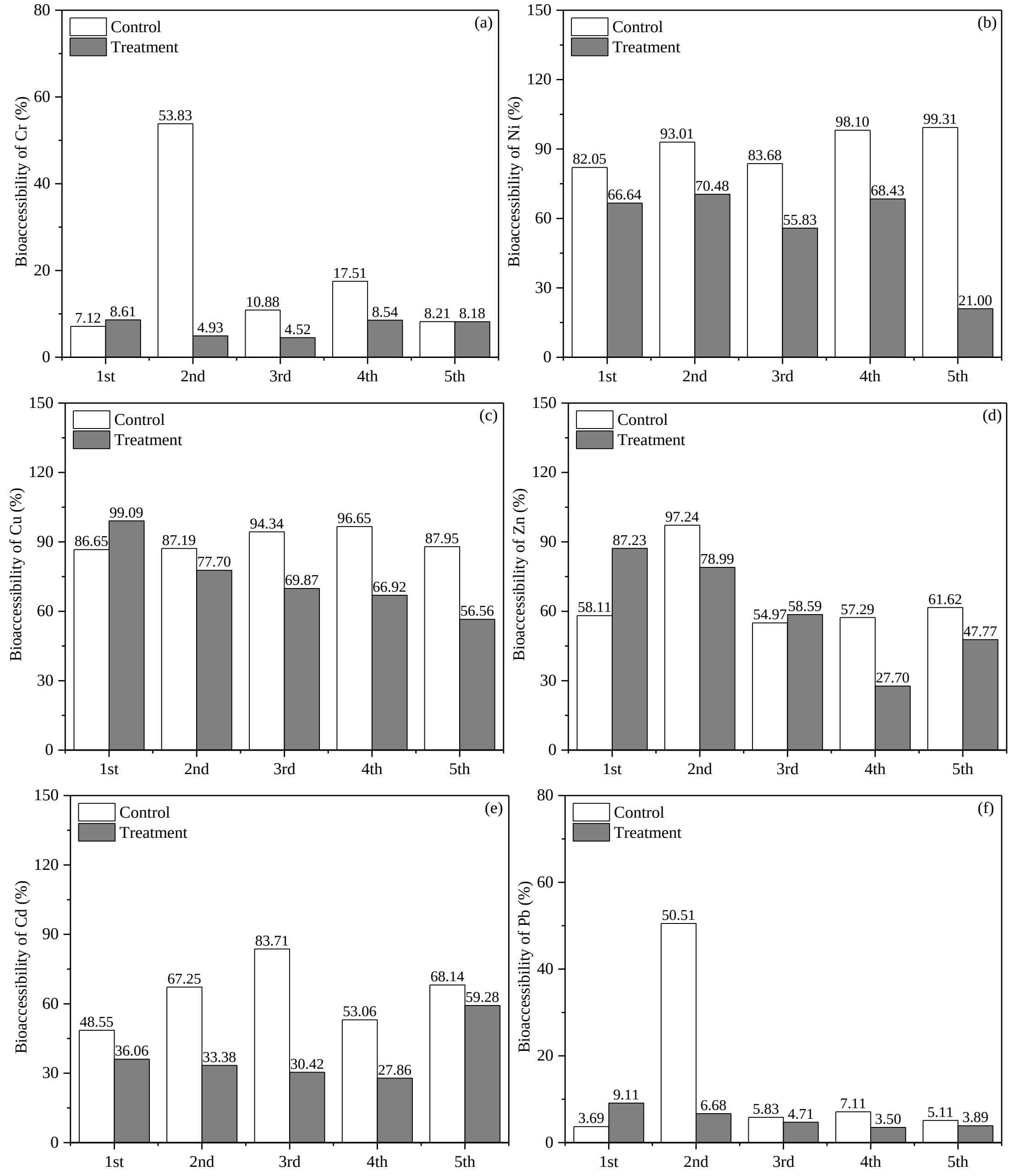

Figure S2. Bioaccessibility (%) of Cr (a), Ni (b), Cu (c), Zn (d), Cd (e) and Pb (f) in the edible portion of the vegetable under simulated gastrointestinal conditions at different harvest times.
